# Effects of Environmental Conditions on High-Yield Magnetosome Production by *Magnetospirillum gryphiswaldense* MSR-1

**DOI:** 10.29252/.23.3.209

**Published:** 2019-05

**Authors:** Leila Hatami-Giklou Jajan, Seyed Nezamedin Hosseini, Masoud Ghorbani, Seyed Fazlollah Mousavi, Behzad Ghareyazie, Mohsen Abolhassani

**Affiliations:** 1 Department of Research and Development, Research and Production Complex, Pasteur Institute of Iran, Karaj, Iran; 2 Department of Microbiology, Pasteur Institute of Iran, Tehran, Iran; 3 Agriculture Biotechnology Research Institute of Iran (ABRII), Agricultural Research, Education and Extension Organization (AREEO), Karaj, Iran; 4 Hybridoma Lab., Department of immunology, Pasteur Institute of Iran, Tehran, Iran

**Keywords:** Conditioned culture medium, Magnetosomes, *Magnetospirillum gryphiswaldense*, MSR-1

## Abstract

**Background:**

Magnetotactic bacteria are a heterogeneous group of Gram-negative prokaryote cells that produce linear chains of magnetic particles called magnetosomes, intracellular organelles composed of magnetic iron particles. Many important applications have been defined for magnetic nanoparticles in biotechnology, such as cell separation applications, as well as acting as carriers of enzymes, antibodies, or anti-cancer drugs. Since the bacterial growth is difficult and the yield of magnetosome production is low, the application of magnetosome has not been developed on a commercial scale.

**Methods:**

*Magnetospirillum gryphiswaldense* strain MSR-1 was used in a modified current culture medium supplemented by different concentrations of oxygen, iron, carbon, and nitrogen, to increase the yield of magnetosomes.

**Results:**

Our improved MSR-1 culture medium increased magnetosome yield, magnetosome number per bacterial cell, magnetic response, and bacterial cell growth yield significantly. The yield of magnetosome increased approximately four times. The optimized culture medium containing 25 mM of Na-pyruvate, 40 mM of NaNO3, 200 µM of ferrous sulfate, and 5-10 ppm of dissolved oxygen (DO) resulted in 186.67 mg of magnetosome per liter of culture medium. The iron uptake increased significantly, and the magnetic response of the bacteria to the magnetic field was higher than threefold as compared to the previously reported procedures.

**Conclusion:**

This technique not only decreases the cultivation time but also reduces the production cost. In this modified method, the iron and DO are the major factors affecting the production of magnetosome by *M. gryphiswaldense* strain MSR-1. However, refining this technique will enable a further yield of magnetosome and cell density.

## INTROUDUCTION

One of the most common Gram-negative prokaryotic cells with heterogeneous characteristics is magnetotactic bacteria being able to produce linear chains of bacterial magnetic particles (BacMPs) called magnetosomes[1]. BacMPs are intracellular organelles composed of magnetic iron particles surrounded individually by a phospholipid bilayer. The size of the magnetosome particles often varies within the species, ranging from 35 to 120 nm[2]. Magnetosomes mainly composed of magnetite (Fe_3_O_4_) or greigite (Fe_3_S_4_) are assembled as one or more chain(s) depending on different elements and typically located close to the cytoplasmic membrane. Meanwhile, the number of magnetosomes in *Magnetospirillum gryphiswaldense* MSR-1 often differs with regard to the environmental conditions[3,4]. Magnetic bacteria have high biomineralization ability and are able to adjust themselves to new environmental conditions such as sever deprivation of metals[5]. It has been suggested that prokaryotes can be classified into biologically induced and biologically controlled mineralization based on their ability in synthesis of minerals[6].

Microbial reduction of metals and formation of magnetosomes within microorganisms in a marine environment were first discovered by Blakemore in 1975[7]. The biological production of the biomineralized magnetosomes is strictly controlled at the gene level, and the magnetosomes are normally formed in different sizes and shapes in magnetosome membrane[6]. Magnetosome synthesis has recently been proposed as a model for the formation of prokaryotic organelles and biomineralization[8,9]. Although the details of the mechanism for the synthesis of magnetosomes are not exactly clear, studies have shown that the formation of magnets is a cellular process that depends on several stages, including the separation of the internal membrane of the cell, the transfer of ions, the crystallization of magnetite within these vesicles, and the formation and arrangement of adult crystals as a linear structure of the cellular skeleton[1,10].

Unlike the chemical synthesis of other nanocrystals, magnetosomes are synthesized via unique features, including a perfect crystallographic appearance, a narrow and single magnetic domain in nanosize range with a permanent magnetization, and the formation of a biocompatible lipid bilayer around each mineral particle[2,11-13], which bring about an exceptional importance in biotechnological applications of magnetic nanoparticles such as nuclear magnetic resonance, cell separation assays as drug carriers, and destruction of tumor cells by hyperthermia[14-18]. Since 1991, several applications including carriers for enzymes[19], nucleic acids[8,20], and antibodies[19] as well as anticancer drugs[8,9,21] have been reported for bacterial magnetosomes. However, because of the difficulty in growing magnetotactic bacteria and the low-yield production of magnetosomes, these applications have not been extended to commercial scale[10,13]. Various kinds of culture media such as the optimized flask medium (OFM), large-scale medium, magnetic spirillum growth medium, and optimized growth medium have been developed to fulfill the requirements of magnetotactic bacteria[11]. Adjustment of oxygen, temperature, and redox potential have been demonstrated to be remarkably effective in magnetosome production and magnetotactic bacterial yield in fed-batch flasks and bioreactors[11,12,22]. Moreover, most of the magnetotactic bacteria strains have been found to consume oxygen, ferric quinate, and nitrate as electron acceptors and use succinate or lactate, acetate, and nitrate as electron donors[11]. Culture medium optimization was very effective for high-yield cultivation of magnetosome, as it has previously been reported that at higher dissolved oxygen (DO) level, cell growth would be greater, but for higher yield of magnetosome, low DO concentrations are the prerequisite[23,24]. Thus, to resolve this situation, it is necessary to enhance DO to an optimum level by stirring the medium to increase the magnetotactic bacteria growth and allow the microbe to lower DO by the respiration process to an optimum level. Some of the important obstacles in the mass culture of magnetic bacteria are adjusting the oxygen level and optimizing the culture medium.

The main aim of this study was to investigate the effect of various concentrations of medium compounds, including oxygen, iron, carbon, and nitrogen to improve the growth of *M. gryphiswaldense* MSR-1 and to increase the yield of magnetosome.

## MATERIALS AND METHODS

### Bacterial strain and culture

*M. gryphiswaldense* MSR-1 was purchased from Leibniz-Institute, DSMZ-German Collection of Microorganisms and Cell Cultures (Germany) and cultured in the initial medium in a 7.5-L fermentor (oxystate, FerMac, 310/60, UK). All chemicals (analytical grade) were procured from Merck (Germany).

### Initial medium preparation

The initial culture medium was prepared with the Na-lactate medium as described previously[10]. Briefly, all medium components, except K_2_HPO_4_, were dissolved in 5.4 L of distilled water in a 7.5-L fermentor and then autoclaved at 121 °C for 30 min. K_2_HPO_4_ was dissolved in 200 ml of distilled water and after sterilization, pumped into the fermentor before inoculation. Optimal culture condition was confirmed by adjusting the types and amounts of iron, nitrogen, and carbon sources. The growth of *M. gryphiswaldense* MSR-1 was evaluated using optical density (OD) at 400 nm, by an ultraviolet-visible (UV-Vis) spectrophotometer (Lambda Bio35, Perkin Elmer, USA).

### Growth conditions

Bacterial cells were cultured in a 7.5-L oxystate fermentor at 30 °C for 50 hours, and pH was maintained at 6.8 during cultivation by the nutrient solutions (ferric quinate, 6.5 g; sodium pyruvate, 159 g; lactic acid, 52.6 g; NaNO_3_ per liter, 84.5 g). Initial air flow and stirring rate were controlled at 0.5 L min-1 and 100 rpm, respectively[12].

### Preparation of optimized medium

The OFM or modified OFM (MOFM) contained 0.1 g of MgSO_4_.7H_2_O, 0.05 g of sodium thioglycolate, 0.1 g of yeast extract, 0.5 g of K_2_HPO_4_, 5 ml of mineral elixir, 200 μM of ferrous sulfate, 40 mM of NaNO_3_, and 25 mM of Na pyruvate, per liter deionized water. The pH was adjusted to 6.8 with NaOH. The inoculum used for the initiation of culture was grown by three sequential transfers at a ratio of 10% (v/v) at 30 °C and agitated in a shaking incubator at 100 rpm for 50 h.

### Determination of growth and magnetite formation

Cell growth was determined at OD 400 nm. The average magnetic orientation of cell suspensions was assayed by an optical method as described previously[25]. Briefly, bacterial cells were aligned at different angles relative to the light beam by means of an external magnetic field using a spectrophotometer (Hitachi U-2800, Japan). The ratio of the resulting maximum and minimum scattering intensities (magnetic response [Cmag]) was shown to be well correlated with the average number of magnetic particles and can be used for the semi-quantitative evaluation of magnetite formation (Cmag = 1 corresponds approximately to 10 particles per cell).

### Isolation of magnetosomes

*M. gryphiswaldense* cells (approximately 10 g of wet weight) was suspended in a 100-ml buffer containing 20 mM of HEPES, 4 mM of EDTA, 0.1 mM of phenylmethylsulfonyl fluoride, pH 7.4 and disrupted three times using an ultra homogenizer (GEA Niro Soavi, Germany). The suspension was then centrifuged at 680 ×g for 10 minutes to remove the unbroken cells, and the cell debris and the supernatant were passed through a MACS magnetic separation column (Germany’s Miltenyi Biotec). To bind the magnetic particles to the column matrix, the columns were placed between two Sa-Co-magnets generating a magnetic field gradient inside the column. The separation of magnetosome particles was completed at the time when there was no trace of black magnetosome-like particles in the cell extract after passage through the column. The attached magnetic particles to the column were rinsed with 50 ml of 10 mM HEPES, 200 mM of NaCl, pH 7.4 and subsequently with 100 ml of 10 mM HEPES, pH 7.4, to eliminate electrostatically bound contamination. The column was then removed from the magnets, and magnetic particles were eluted from the column by flushing out with a 10-mM HEPES buffer. The magnetosome suspension was finally loaded on the top of a sucrose cushion (55% [wt/wt] sucrose in 10 mM HEPES, pH 7.4) and centrifuged in a swinging 50-ml bucket rotor at 280,000 ×g at 4 °C for 8 hours using an ultracentrifuge. The supernatant containing sucrose ingredient was removed, and the pelleted magnetosomes were suspended in 0.1-M PBS buffer (pH 7.4) and centrifuged at 11,000 ×g at 4°C for 8 minutes, finally disrupted using an ultrasonic cell crusher (300 W) for 4 s with an interval of 8 s and repeated 80 times. Extracted magnetosomes were then absorbed to the bottom of the beaker using a magnetic field, and the cell debris was removed by discarding the supernatant. The magnetosomes were resuspended in 0.1 M of PBS (pH 7.4) and ultrasonically cleaned (45 W, for 4 s with an interval of 8 s and repeated 40 times) and collected. All the processes were repeated 15 times. The purified magnetosomes were finally dried using a vacuum freeze-drying method (Kinetics, EZ550Q) and kept at 105 °C for 24 h and weighed. The purified magnetosomes were sterilized by Co60 irradiation (15 kGy) and resuspended in 0.1 M of PBS (pH 7.4) at a concentration of 500 μg/mL[26].

### Isolation of nonmagnetic subcellular fractions

After separation of magnetosomes, an aliquot of the cell extract was subjected to ultracentrifugation (330,000 × g, at 4°C, 1 h). The supernatant contained the soluble proteins, and the pellet contained membrane fraction that was further separated by isopycnic centrifugation as described by Osborn and Munson[27].

### Iron concentration

Iron concentration was measured by an atomic absorption spectrometer (Model Z8000; Hitachi, Japan) using an acetylene/air flame, as described previously by Heyen and Schüler[28].

### Transmission electron microscopy (TEM)

Bacterial cells in the pellet were rinsed three times, suspended in distilled water, adsorbed onto a 300-mesh carbon-coated copper grid and viewed directly by a transmission electron microscope (Philips Tecnai F 30, FEI Company, USA) at an accelerating voltage of 300 kv for recording magnetosomes.

### Measuring of magnetosome yields

The magnetosome yield in bacterial cell was determined by four different approaches as: (1) counting the magnetosome numbers in at least 50 bacterial cells in TEM pictures, (2) measurement of the total magnetosome weight extracted directly from the bacterial cell culture, (3) indirect measurement of magnetic response of magnetosomes in each bacteria cell, and (4) measurement of intracellular iron uptake as explained above.

### Statistical analysis

The data were statistically evaluated using ANOVA at the significance level of *p* < 0.05 using GraphPad Prism software (v 7.01).

## RESULTS

To achieve a high yield of magnetosome by *M. gryphiswaldense* strain MSR-1, we modified the composition of the initial culture medium using 25 mM of sodium pyruvate instead of sodium lactate as the carbon source and electron donor, as well as using 40 mM of sodium nitrate instead of ammonium chloride as the nitrogen source. The medium was also contained 200 µM of ferrous sulfate instead of 100 µM ferric citrate as a major source of iron. As the electron acceptor, O_2_ concentration was kept at 5-10 ppm at all times by continuous injection. The incubation temperature was set at 30 °C, and the pH was kept at 6.8. In all the experiments, the initial concentration of *M. gryphiswaldense* was 1.05 × 10^8^/ml.

### Effect of oxygen concentration

**Fig. 1:**
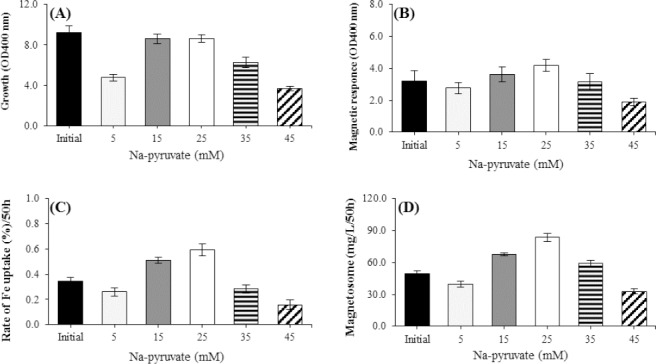
Effects of dissolved oxygen (DO) concentration on factors affecting magnetosome production by *M. gryphiswaldense* MSR-1 during culture in optimized flask medium (OFM; 50 h). (A) Cell growth, (B) Cmag, (C) iron uptake, and (D) the magnetosome production. The maximum rates of cell growth, Cmag, iron uptake, and magnetosome were produced under microaerobic condition (5-10 ppm O_2_). The optimal DO concentration was 5-10 ppb O_2_ (microaerobic) for producing the highest quantity of magnetosome; magnetosome was produced approximately two times more than initial medium (D) (*p* < 0.01). OFM was used as an initial culture medium.

To determine the optimal oxygen concentration during the magnetosome production, three different concentrations were used. *M. gryphiswaldense* MSR-1 was cultured in an anaerobic condition, microaerobic condition with 5-10 ppm oxygen, and an aerobic condition in the initial medium, since most of the magnetic bacteria are often obligate microaerophiles, anaerobes, or both. Our results showed that *M. gryphiswaldense* MSR-1 had the highest growth rate in microaerobic condition, as compared to the aerobic and anaerobic conditions. A rapid increase in DO concentration from microaerobic condition with 5-10 ppm oxygen to aerobic condition in the growth conditions brought about a sharp decrease in the cellular magnetotactic response (Cmag), production of magnetosome, and reduction of Fe uptake. The results also indicated that an optimum concentration of oxygen (5-10 ppm) in microaerobic condition increased the magnetosome production significantly by 43%, as well as Cmag and the rate of Fe uptake significantly increased by 28% and 39%, respectively (Fig. 1B, 1C, and 1D). No significant correlation was observed between the concentration of the oxygen and growth of the magnetic bacteria in the microaerobic and anaerobic conditions. Growing *M. gryphiswaldense* MSR-1 in a growth medium containing 15 mM of sodium lactate as the carbon source, 20 mM of NH_4_Cl sodium lactate as the carbon source, 20 mM of NH_4_Cl as the nitrogen source, and 100 µM of ferric citrate as the iron source at 30 °C (pH 6.8), and a microaerobic condition with 5-10 ppm oxygen resulted in the highest magnetosome yield of 84.23 mg/L, a significant Fe uptake of 0.58%, and the cellular magnetotactic response at 4.26 (Fig. 1B-D). The magnetosome formation was very sensitive to the changes of the oxygen concentration. The optimal concentration of DO in the culture medium was very important environmental factor and played a role in bacteriological magnetosomes and in bacterial growth (Fig. 1).

### Effect of different sources of iron

In order to obtain the optimal iron conditions for the high yield of magnetosome production, the experiments were performed and analyzed in the presence of 100 µM of three different iron sources such as ferric quinate, ferrous sulfate, and ferric citrate separately in the initial medium.

### The effects of iron sources on magnetosome production

**Fig. 2:**
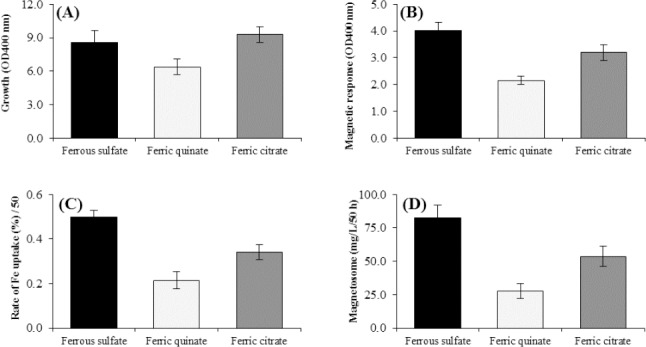
Effect of different iron sources on factors affecting magnetosome production of *M. gryphiswaldense* MSR-1 during culture in optimized flask medium (OFM); 50 h). (A) Cell growth, (B) Cmag, (C) iron uptake, and (D) the magnetosome production. The maximum Cmag, iron uptake, and magnetosome production was obtained in the presence of 100 µM of ferrous sulfate versus the other iron sources. The highest amount of magnetosome was produced in the presence of the ferrous sulfate, which was higher by 79% and 35%, respectively as compared with ferric quinate and ferric citrate (D) (*p* < 0.05). It is notable that ferrous sulfate and ferric citrate as iron sources showed no significant effect on cell growth (A).

The effects of ferrous sulfate on the cell growth, magnetic response, rate of Fe uptake, and magnetosome yield were compared with other sources of iron such as ferric citrate and ferric quinate in the presence of optimal oxygen concentration. As Figure 2 shows, the cell growth was significantly higher in the presence of ferrous sulfate or ferric citrate as compared with ferric quinate (Fig. 2A), whereas there was no significant difference between ferrous sulfate and ferric citrate. However, ferrous sulfate was significantly superior in increasing the magnetic response and magnetosome production as compared with ferric citrate (by 35%) and ferric quinate (by 70%), as represented in Fig. 2C and 2D. The rate of Fe uptake was significantly higher in the presence of ferrous sulfate as compared with two other sources of iron in the medium (Fig. 2B).

### Effects of ferrous sulfate concentrations on magnetosome production

**Fig. 3:**
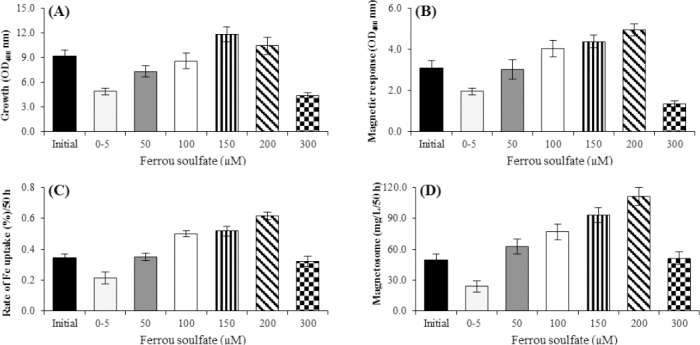
Effect of different concentrations of ferrous sulfate on the factors affecting magnetosome production by *M. gryphiswaldense* MSR-1 during culture in optimized flask medium (OFM; 50 h). (A) Cell growth, (B) Cmag, (C) iron uptake, and (D) the magnetosome production. The maximum rate of cell growth in the concentration of 150 μM (A) and the maximum rates of Cmag, iron uptake, and magnetosome production were obtained in the presence of 200 μM ferrous sulfate (B, C, and D). The optimum concentration of ferrous sulfate for the highest magnetosome production yield was determined to be 150 to 200 μM. At these concentrations, the magnetosome production was 62% more than the initial condition (D) (*p* < 0.01).

Cells were cultured in the presence of 5, 50, 100, 150, 200, and 300 μM of ferrous sulfate for 50 hours (Fig. 3). At the concentration of 200 μM (111.34 mg/L), both the Fe uptake (as compared to the initial condition of ferric citrate) and the Cmag level increased significantly by 55% and 63%, respectively (Fig. 3B-D), whereas, no significant cell growth was found at this concentration (Fig. 3A). Meanwhile, at lower concentrations of ferrous sulfate (5-100 µM), the growth rate of *M. gryphiswaldense* MSR-1 increased significantly (*p* < 0.05; Fig. 3A). When the ferrous sulfate concentration was over 200 µM, a significant decrease was observed in bacteria cell growth. Ferrous sulfate is believed to be essential for the synthesis of magnetosomes (Fig. 3A).

### Effects of different sources of carbon

Four different common carbon sources, including sodium pyruvate sodium L-lactate, sodium acetate, and sodium succinate, were investigated in the culture medium (data not show). Sodium pyruvate and sodium L-lactate increased the cell growth, the cellular magnetotactic response (Cmag), the rate of Fe uptake, and magnetosome production significantly, as compared with sodium succinate and sodium acetate (data not show). The results also revealed that sodium pyruvate was even more effective on increasing the magnetosome production (data not shown).

### Effects of different concentrations of sodium pyruvate

**Fig. 4:**
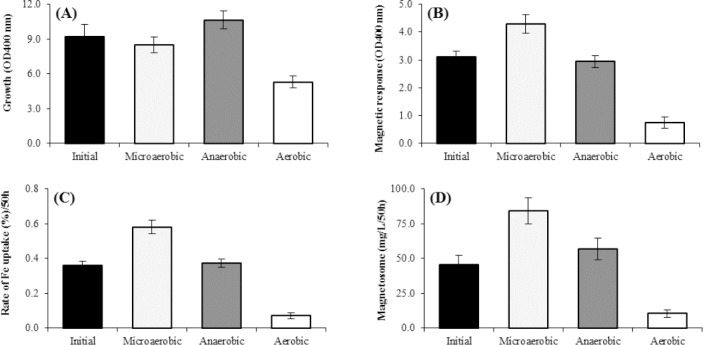
Effect of different concentrations of Na-pyruvate on factors affecting the magnetosome production of *M. gryphiswaldense* MSR-1 during culture in optimized flask medium (OFM; 50 h). (A) Cell growth, (B) Cmag, (C) iron uptake, and (D) the magnetosome production. There was no significant difference in the cell growth at the concentration of 15 and 25 mM of Na pyruvate as compared to the initial condition (A). The Cmag, iron uptake, and magnetosome produced at the concentration of 25 mM of Na pyruvate. Under the optimal concentration of Na pyruvate (~25 mM), the amount of magnetosome was 67%, which was much more than the initial condition (D) (*p* < 0.01).

Five different concentrations of sodium pyruvate in the range of 5, 15, 25, 35, and 45 mM were investigated (Fig. 4). As the sodium pyruvate concentration increased from 5 to 15 mM, the cell growth increased up to 45%. Also, a significant increase was observed in Cmag, the rate of Fe uptake, and magnetosome production (Fig. 4B-D, respectively). However, when the concentration exceeded over 15 mM, the cell growth no longer increased (Fig. 4A). It should be noted that the maximum magnetosome production value was found to be 93.5 mg/L at 50 h when the sodium pyruvate concentration was 25 mM in OFM, which increased 67% in comparison to the control condition (Fig. 4D).

### Effects of different sources of nitrogen

**Fig. 5.**
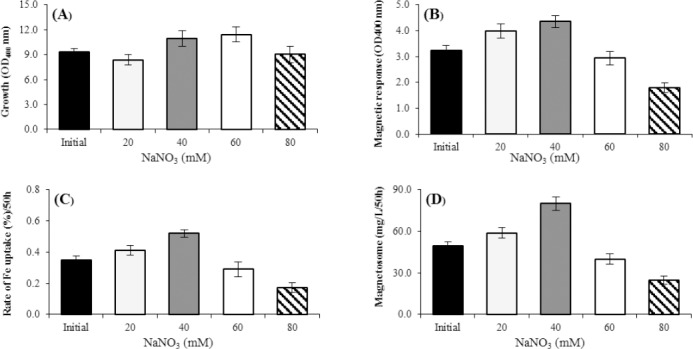
Effect of NaNO_3_ concentrations on magnetosome production in optimized flask medium (OFM; 50 h). (A) Cell growth, (B) Cmag, (C) iron uptake, and (D) the magnetosome production. The maximum cell growth was observed in the concentration of 60 mM NaNO_3_ (A), and the maximum production of Cmag, iron uptake, and magnetosome was under the concentration of 40 Mm NaNO_3_ (B, C, and D) in OFM. The optimal concentration of NaNO_3_ was determined to be 40 mM for the highest magnetosome production yield, which showed 38% more magnetosome production than the initial condition (D) (*p* < 0.01).

Three common nitrogen sources recommended in most of the culture media include NaNO_3_, (NH_4_)_2_SO_4_, and NH_4_Cl with the concentration of 20 mM in OFM (data not shown). The results showed that NaNO_3_ increased the cellular magnetotactic response (Cmag), the rate of Fe uptake, and magnetosome production significantly, as compared to NH_4_Cl and (NH_4_)_2_SO_4_ (data not show). The maximum rate of growth was seen in the presence of NH_4_Cl (data not show). The influence of different concentrations of NaNO_3_ (20, 40, 60, and 80 mM) on growth and magnetosome formation was investigated. By increasing the NaNO_3_ concentration from 20 to 60 mM, the growth rate increased by 27%, while the Cmag, the rate of iron uptake, and the magnetosome production decreased (Fig. 5). At the concentration above 60 mM, no significant changes was observed in the growth rate. At 40 mM, the magnetosome production increased 38% (79.99 mg/L in 50 h; Fig. 5D)

### Optimized conditions for cell growth and magnetosome production

**Fig. 6:**
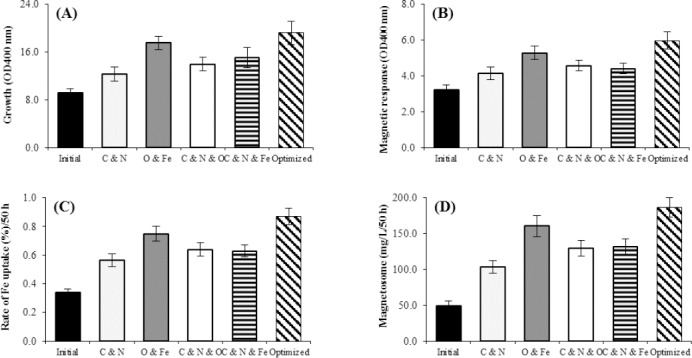
The growth of *M. gryphiswaldense* MSR-1 under different conditions of the types and amounts of iron, nitrogen, and carbon sources and oxygen concentration. The maximum rate of cell growth in the optimized conditions (A), the maximum rate of Cmag, iron uptake, and magnetosome production under optimized oxygen and iron concentration (B, C, and D), respectively. The highest amount of magnetosome was achieved at the optimized condition including optimized oxygen and iron concentrations when magnetosome production was 75% and 64%, respectively (D) (*p* < 0.01).

The growth of *M. gryphiswaldense* MSR-1 under different conditions of the types and amounts of iron, nitrogen, and carbon sources and oxygen concentration is shown in Figure 6. The optimized culture medium, contained 25 mM of sodium pyruvate, 40 mM of NaNO_3_, 200 µM of ferrous sulfate, and 5-10 ppm of O_2_ concentration. The highest magnetosome production was about 186.87 mg/ L/50h (Fig. 6).

### Tranmission electron microscopy of magnetosom

**Fig. 7:**
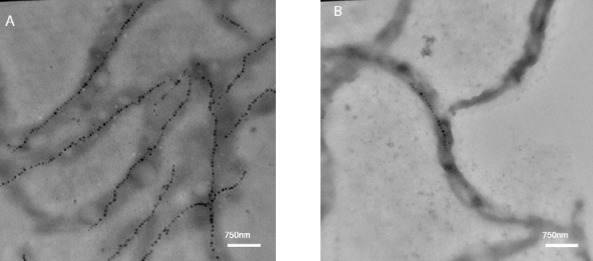
Transmission electron microscopy image of magnetosome chains of *M. gryphiswaldense* MSR-1. (A) Under the optimized culture medium, and (B) under the initial culture medium.

In order to prove the high level of magnetosome production in the optimized conditions and initial in 50 h, TEM was performed (Fig. 7).

## DISCUSSION

In this report, we attempted to increase the production of magnetosome in MSR-1 strain of *M. gryphiswaldense* by optimizing the supply of oxygen, iron, carbon, and nitrogen. The results showed that the oxygen has a critical role in the synthesis of magnetosomes. At DO level above 5-10 ppm, the iron uptake and magnetosome production reduced, but the bacterial growth was normal. However, at lower DO (<5-10 ppm), the rate of iron uptake, Cmag, and magnetosome production increased that is likely associated with the slow growth of bacteria. These observations are in agreement with those reported by Yang *et al*.[13]. It has also been reported that magnetosome yield could be significantly increased under microaerobic conditions; however, the exact role of oxygen in magnetosome biomineralization is still unclear[11]. Most reports have claimed that oxygen is necessary to maintain the required redox potential for magnetosome growth[12,28]. *M. gryphiswaldense* MSR-1 is facultative anaerobic bacteria with aerobic condition preference for the cell growth[13]. During magnetotactic bacteria growth the concentration of O_2_ had a specific effect on the synthesis of magnetosomes. It increases the magnetosome production at concentrations lower than 5-10 ppm, whereas at higher concentrations, it decreases the magnetosomes[4,29,30]. Therefore, controlling DO level in the culture medium or altering aerobic/anaerobic conditions is favorable for magnetosome production[4,25,31-33].

In addition to oxygen levels, our study showed that the iron source is another important factor for more efficient uptake of this vital ion and the magnetosome production. Ferrous sulfate was better source of iron for *M. gryphiswaldense* than ferric quinate and citrate, perhaps because the reduced form of ferrous is more soluble compared to the oxidized analogue. This result is in line with the findings of Yang *et al*.[24].

We showed that the excess concentrations of iron in the culture medium can be toxic for *M. gryphiswaldense* MSR-1. A slight increase in extracellular iron concentration elevated the iron uptake, magnetosome production, Cmag, and cell growth, but the ferrous sulfate concentration above 150 μM led to reduction in the cell growth and enhancement of the iron uptake rate, Cmag, and magnetosome production. Iron concentrations ≥300 μM could damage the bacteria seriously and significantly reduce the rate of iron uptake, Cmag, magnetosome production, and the growth rate (Fig. 3). In magnetotactic bacteria, iron not only acts as a protein cofactor but also accelerates the biomineralization process in the cells[6,14,29]. It has been demonstrated by Faivre *et al*.[29] that *M. gryphiswaldense* utilizes soluble ferrous ions and ferric (ferritin form) synchronously for the magnetosome production. Maximum magnetosome production was observed at 200 μM of iron, which was in agreement with the reported results[34-36]. Using TEM analysis, we proved that in the optimized culture medium, both the quantity and size of magnetosome increased (Fig. 7). This observation was also noted by Liu *et al*.[12]. Furthermore, the increased number of double chains in bacteria indicates the optimal medium is an appropriate condition for the growth of *M. gryphiswaldense* (Fig. 7).

Our results confirmed the superiority of pyruvate and lactate for magnetosome production over acetate and succinate as reported previously[12,28]. This preference may indicate that pyruvate and lactate have better redox potential in comparison with succinate and acetate as the electron donor for ATP synthesis[37]. We showed that 25 mM sodium pyruvate was the optimal concentration for magnetosome production by *Magnetospirillum sp*., as reported also by Liu *et al*.[12] and Zhang *et al*.[22]. They concluded that such behavior can be related to the bacterial susceptibility to increased osmotic potential[22]. For magnetosome synthesis, the role of nitrogen is very important to form proteins for the assembly of magnetosome and to transport and incorporate iron in the magnetotactic bacteria. Our data showed that in the *magnetotactic* bacteria, the use of NaNO_3_, as a nitrogen source, leads to more magnetosome production versus using NH_4_Cl and (NH_4_)_2_SO_4_. This result contradicts the results of Liu *et al*.[12]. Nitrate is a strong oxygen acceptor having redox potential, which supports our observations (Fig. 6). The culture medium containing the low concentration of nutrients (in particular carbon and nitrogen) is a key limiting factor that affects magnetosome production and cell density of all magnetotactic bacteria. Our results showed that in magnetosome synthesis, the rate of iron uptake and Cmag enhanced at lower concentrations of NaNO_3_ (40 mM), as compared to the higher concentrations (60 mM), which support previous results[38]. Proteins promote the nucleation of iron crystals, leading to magnetosome production in magnetotactic bacteria after the vesicles assembling[39]. It has recently been illustrated that the magnetosome-associated membrane proteins play a significant role in magnetosome crystals growth inside the magnetotactic bacteria[39,40]. Our result showed that within 50-h culture, a maximum amount of magnetosome was produced (about 186.87 mg L^−1^), and 5.76 g L^−1^ bacterial cells were obtained, whereas the cell growth rate was 2.768 g L^−1^/day.

The magnetic nanoparticles are synthesized in the presence of low oxygen and high level of iron concentrations under the influence of intracellular microbial reduction as trivalent iron compounds[4,41]. The numbers and shapes of the magnetosomes vary depending on the conditions of the culture medium, in particular, the concentration of available soluble iron, DO, nutrients, temperature, pH, and degradation power. On average, between 10 and 30 magnetosomes are synthesized in each bacterial cell.

For the synthesis of magnetosomes, the bacterial cells need to uptake a large amount of iron from its surroundings and put in the bio-mineralization process[11,38]. Also, these crystals are required to be in well-defined numbers, shapes, and crystal size, in order to play an efficient magnetic field sensor role. The composition of additives in culture medium can have significant effects on the size and other magnetic properties of these nanoparticles[28].

In summary, we report an improved MSR-1 culture medium condition for increased magnetosome yield, magnetosome number per cell, magnetic resonance, and cell yield in a shorter time and the reduced cost. Our method allows achieving the mass production of magnetosomes by MSR-1 in a fermentor scale by choosing the suitable sources and concentrations of culture medium composition. The most important advantages of this protocol are: (1) the concentrations of oxygen, iron, carbon, and nitrogen sources in the medium can be auto-controlled at a constant level by pH-stat feeding, leading to ease of manipulation and elimination of the possibility of nutrient exhaustion during the culture process and (2) easy up-scale process for industrial production without need for genetic manipulation. The DO and iron are the major factors affecting the magnetosome production in the bacterial culture. Further refinements of this protocol to overcome other shortages of the magnetosome production of magnetotactic bacteria are ongoing in our laboratory.
